# The sixth Japanese meeting on biological function and evolution through interactions between hosts and transposable elements

**DOI:** 10.1186/s13100-023-00310-9

**Published:** 2023-12-12

**Authors:** Kenji Ichiyanagi, Yoko Ikeda, Kuniaki Saito

**Affiliations:** 1 Laboratory of Genome and Epigenome Dynamics, Department of Animal Sciences, Graduate School of Bioagricultural Sciences, Nagoya University, Furo-cho, Chikusa-ku, Nagoya, 464-8601 Japan; 2 Institute of Plant Science and Resources, Okayama University, 2-20-1 Chuo, Kurashiki, 710-0046 Japan; 3 Invertebrate Genetics Laboratory, National Institute of Genetics, Yata 1111, Mishima, Shizuoka, 411-8540 Japan

**Keywords:** Transposon, Retrotransposon, Epigenetics, Gene innovation, Evolution

## Abstract

The sixth Japanese meeting on host–transposon interactions, titled “Biological Function and Evolution through Interactions between Hosts and Transposable Elements,” was held on August 24th and 25th, 2023, at the National Institute of Genetics as well as online. This meeting was supported by the National Institute of Genetics and aimed to bring together researchers studying the diverse roles of TEs in genome function and evolution, as well as host defense systems against TE mobility, TE bursts during evolution, and intron mobility in mammals, insects, land plants, yeast, protozoa, and bacteria. Here, we have presented the highlights of the discussion.

Organizers: Kenji Ichiyanagi, Yoko Ikeda, and Kuniaki Saito.

## Introduction

Transposable elements (TEs) constitute a significant proportion of the eukaryotic genome and play key biological and evolutionary roles. The interactions between TEs and their hosts were discussed in a series of meetings titled “Biological Function and Evolution through Interactions between Hosts and Transposable Elements” [1–3]. This meeting was held at the National Institute of Genetics (NIG), Mishima, Japan, where biologists studying TEs in different species gathered and exchanged ideas. At the sixth meeting, held on August 24th and 25th, 2023, we invited 17 speakers to present their recent works in six sessions chaired by Akira Kanazawa (Hokkaido University, Japan), Tetsuji Kakutani (University of Tokyo, Japan), Yoichi Shinkai (RIKEN, Japan), Mari Ohnuki (Kyoto University, Japan), Yota Murakami (Hokkaido University, Japan), and Haruhiko Siomi (Keio University, Japan). The meetings had approximately 50 onsite attendees and 40 online attendees.

## Highlights of the talks

### Epigenetic regulation of TEs

Epigenetic regulation involving DNA methylation, histone modification, and small RNA pathways is a pivotal mechanism for silencing the transcription and mobility of TEs in eukaryotes. Taiko Kim To (Tokyo Institute of Technology, Japan) introduced recent findings on a novel de novo DNA methylation pathway named RdDM-independent CH methylation Establishment (RiCHE) [4]. In many plant species, the silencing of heterochromatin depends on H3K9 methylation and DNA methylation of CpG and non-CpG sequences (or CpH; H stands for A/T/C). The establishment of DNA methylation in both sequences depends on an RNAi-based pathway called RNA-directed DNA methylation (RdDM). However, paradoxically, deficiency in the RdDM mechanism results in hypomethylation in euchromatin regions rather than in heterochromatin regions. Therefore, it is unclear how heterochromatin marks are targeted to specific regions. Dr. To presented recent epigenomic analyses [5, 6], indicating that a novel pathway designated as RiCHE can strongly and precisely induce de novo methylation in both the CpH sequence and histone H3K9 in most TEs. Interestingly, this pathway prefers the coding regions of TEs, and its specificity has been suggested to involve histone H2A variants. Furthermore, the establishment of RNAi-independent CpH/H3K9 methylation is dependent on CpG methylation, indicating that crosstalk among epigenetic modifications drives heterochromatin formation in *Arabidopsis*. Yoko Ikeda (Okayama University, Japan) presented a DNA methylation regulatory mechanism in the liverwort, *Marchantia Polymorpha*, which is a member of a basal land plant lineage. Flowering plants have evolved a system for maintaining stable CpG methylation in the next generation. In contrast to that in mammals, active DNA demethylation does not occur in plant germ cell lines. However, in Marchantia, genome-wide changes in both CpG and non-CpG methylation were observed, suggesting the presence of different regulatory mechanisms for DNA methylation. MpMET, an ortholog of the maintenance CpG methylation enzyme, and two copies of orthologs of DNA demethylation enzymes were found in the Marchantia genome [7]. Based on the disruption mutant analysis, Dr. Ikeda and her colleagues found that these contribute to DNA methylation and TE regulation through unique features of Marchantia. Eriko Sasaki (Kyushu University, Japan) discussed the genetic architecture underlying DNA methylation variation (i.e., epigenetic diversity) and its role in regulating TEs in natural populations of *Arabidopsis thaliana*. Although this variation is often referred to as the epigenome and is speculated to result from past environmental effects, Dr. Sasaki and her colleagues have demonstrated that a significant portion of this variation is influenced by genetic variation in *trans* modifiers, especially epigenetic regulators including *CHROMOMETHYLASE2* (*CMT2*), *CMT3*, and *NRPE1*, the largest subunit of RNA polymerase V [8]. She also showed that the novel regulator *JMJ26* specifically controls CHG methylation in RdDM-targeted TEs. These alleles displayed a geographic cline and were strikingly associated with the regional climate, suggesting a form of local adaptation. Although the specific nature of this adaptation remains unclear, the results of population genetics provide evidence that combinations of *trans*-modifiers influence the mobilization of TEs by controlling DNA methylation levels under natural conditions.

In budding yeast, *Saccharomyces cerevisiae*, a long terminal repeat (LTR) retrotransposon named Ty1 exhibits an interesting characteristic regarding the regulation of transcription. Hiroshi Masumoto (Nagasaki University, Japan) demonstrated the silencing effect of Ty1, which has a sequence that can silence a transgene inserted between *gag* and *pol* [9]. Dr. Masumoto and his colleagues recently found that the transcription fork originating from the 5′-LTR promoter passes through the insertion region, canceling the silencing effect and allowing expression of the inserted gene. In contrast, the silencing effect is restored when the transcription fork originating from the 5′-LTR is stopped. This sequence region exerted a silencing effect in only one direction and did not interfere with the generation of transcription forks from the 5′-LTR upstream. This makes it a unique silencing region, in which the silencing effect can be switched on and off by a transcription fork.

In animals, PIWI-interacting RNAs (piRNAs) play an important role in TE silencing in germ and gonadal somatic cells. These RNAs are generated from precursor transcripts. However, the evolutionary origin of piRNA source loci, called piRNA clusters, which are dispersed over TE-accumulated regions in the genome, remains elusive. Keisuke Shoji (University of Tokyo, Japan) presented a model for the origin of piRNA clusters in insects. His colleagues first identified a piRNA cluster named “*torimochi*” in BmN4 cells from silkworm ovaries [10]. Using an updated silkworm genome, Shoji et al. found that *torimochi* is a complete transposon with LTRs. Its transposition activity in BmN4 cells, assessed using a nanopore sequencer, was the highest among all the TEs. When the cells were in vitro differentiated into non-gonadal cells (i.e., adipocytes), the expression of *torimochi* decreased, whereas other TEs were derepressed. Taken together, they proposed that *torimochi*, an unfragmented active transposon, integrates with a BmN4-specific activation mechanism and serves as a model for nascent piRNA cluster formation [11].

### Anti-TE mechanisms at the protein and DNA levels

TE activity is restricted by mechanisms other than epigenetic regulation. Kuninori Suzuki (University of Tokyo, Japan) demonstrated that selective autophagy downregulates Ty1 retrotransposition in budding yeasts. Autophagy is induced under starvation conditions and plays a cytoprotective role by degrading unwanted cytoplasmic components. Ty1 is the most abundant retrotransposon in *S. cerevisiae* and introduces mutations via retrotransposition. Retrotransposition intermediates are found in virus-like particles (VLPs), which are localized in the cytoplasm. Interestingly, Atg19-dependent selective autophagy eliminates Ty1 VLPs from the cytoplasm under starvation. In the absence of Atg19, the Ty1 transposition frequency continually increased during starvation [12]. This suggests that selective autophagy safeguards genomic integrity by suppressing TE mobility during nutrient starvation in eukaryotic cells.

Binuclear protozoans, ciliates, whose lineage is evolutionarily distant from animals or plants, also use epigenetic marks to transcriptionally silence TEs; however, they also eliminate the DNA sequences of marked TEs from their active genome. The ciliate *Tetrahymena thermophila* possesses two distinct nuclei within a single cell: the transcriptionally inactive germline micronucleus (MIC) and the transcriptionally active somatic macronucleus (MAC). During sexual reproduction, the MIC differentiates into the MAC of sexual progeny. In this process, more than 12,000 loci containing TEs and their remnants are heterochromatinized de novo and eventually eliminated from the MAC genome. Kensuke Kataoka (National Institute for Basic Biology, Japan) studied the dynamics of heterochromatin and its effects on DNA elimination [13, 14]. Kataoka et al. found seven HP1-like proteins that accumulate with TEs before their elimination, and a subset of these HP1 proteins plays a central role in DNA elimination. These HP1 proteins physically interact with each other and cooperatively recognize histone H3 methylated at K9 or K27, whose genomic locations are dictated by small RNA pathways. These findings suggest that in addition to small RNA-directed TE recognition, the cooperative action of multiple HP1-like proteins ensures robust DNA elimination of thousands of TE-related sequences in *Tetrahymena*.

### TE burst in plants

In some species, certain TEs are amplified rapidly (TE burst), which provides their hosts with an opportunity to significantly change their phenotype. The genus Vigna is a reservoir of diversity consisting of more than 100 species, many of which have adapted to harsh environments, including coastal, desert, marsh, and limestone karst [15]. Given that most Vigna species have a small diploid genome (2n = 22, 500–600 Mb in haploids), the genus Vigna can serve as a model for comparative genomic studies to elucidate the evolution of responses to abiotic stresses. Using a long-read sequencer, Ken Naito (National Agriculture and Food Research Organization, Japan) determined the genomes of 12 Vigna species, including *V. marina* and *V. riukiuensis*, that have adapted to coastal environments and acquired salt tolerance. The scaffolds obtained showed that the overall structure of the genome, including gene order, was highly conserved [16]. However, subsequent ortholog analysis identified very few cases of copy number variations in stress-related genes, which are often reported in species adapted to extreme environments. GO-enrichment analysis on species-specific orthogroups revealed that *V. marina* and *V. riukiuensis* have accumulated many copies, with *V. riukiuensis* having more than 1000 copies, of WUSCHEL-related homeobox (WOX) gene family. The amplified WOX genes in the two species were mostly surrounded by identical LTR sequences at both ends, with typical target site duplications (TSDs). Although the contribution of WOX gene amplification to coastal adaptation remains unknown, their study revealed that LTR retrotransposons can burst rapidly and have the potential to affect gene content in certain species.

Eigo Fukai (Niigata University, Japan) and his colleagues have been working on TEs in the model legume *Lotus japonicus*. They found recent retrotransposition events of several LTR retrotransposons in three recombinant inbred-line populations (RILs) of *L. japonicus* [17]. These LTR retrotransposons remained silenced for a long time in the parental accessions, suggesting that they were epigenetically activated by hybridization and subsequent selfing during the establishment of RILs. Although the molecular mechanism of this phenomenon is still unknown, their observations argue in favor of a hypothesis that hybridization can induce “genomic shock,” which causes transgenerational TE derepression and consequent TE amplification, even if the parents do not have strong reproductive barriers.

Yuki Mizunaru (Kyushu University, Japan) discussed the transposition of *Tpn1* family transposons (*Tpn*) belonging to the *CACTA* superfamily in Japanese Morning Glory (*Ipomoea nil)*. Transposition of the *Tpn1* family frequently accounts for most mutations affecting the phenotype. However, most genomic *Tpn* copies are nonautonomous elements that require a transposase encoded by an autonomous element for transposition [18]. *TpnA2*, one of the autonomous elements, is present in only one copy in Asian strains including Japan, and maintains the transposase gene stably due to deletion of the 3′ terminal region [19]. Genetic experiments have shown that *TpnA2* is a major transposase source. In Japan, numerous mutants have been recorded since the middle of the eighteenth century, suggesting that such mutations are caused by the activation of *Tpn* transposition. Analysis of the genomic sequences of 140 Japanese strains revealed that, in *hypermutable* strains, the number of *Tpn* copies has increased. Most of these strains share mutations caused by *Tpn* transposition in two flower color genes, suggesting that they were derived from a single plant with increased *Tpn* transposition activity. Taking advantage of the homogeneity of the genome, Mizunaru et al. identified candidate genes for *Tpn* activation in Japanese strains by comparing the genome data between two groups showing activated and silenced *Tpn* transpositions.

### TE functions as a protein and DNA

TE sequences are known sources of novel protein-coding genes. So Nakagawa (Tokai University, Japan) has discussed the diversity and evolution of de novo protein-coding genes derived from endogenous virus-like elements (EVEs), including endogenous retroviruses (ERVs). Several protein-coding EVEs, primarily ERVs, are present in the genomes of various mammalian species. Most of these encoded proteins are truncated owing to nonsense and/or frameshift mutations and are epigenetically silenced. However, some are under purifying selection, suggesting that they provide new functions to their hosts, such as cell-cell fusion activity involved in placental development [20]. However, it is difficult to determine whether EVE-derived ORFs function in the host. For example, although the ORF remains intact, they recently found that syncytin-2, which is involved in placental development in humans, does not exhibit fusogenic activity in various Platyrrhini (i.e., New World monkeys) [21]. To investigate functional protein-coding EVE-derived genes (> 80 amino acids with viral protein motif(s)), Dr. Nakagawa his colleagues constructed a database named gEVE (http://geve.med.u-tokai.ac.jp) of 19 mammalian genomes [22]. Various high-quality mammalian genomes have been sequenced. Thus, updating the gEVE database will provide a foundation for studying the specialization and evolution of mammalian species. For example, Nakagawa and his colleagues found, in the echidna genome, an ERV envelope-derived sequence showing a fusogenic activity [23]. Koichi Kitao (Nagoya University, Japan) presented his research on the search for protein-coding genes derived from LTR retrotransposons, including ERVs, in the genomes of monotremes, birds, and reptiles. His group first identified ERV-derived protein-coding genes, designated as RTOM1, 2, and 3, by comparative genomic analyses of two egg-laying mammals: the platypus (*Ornithorhynchus anatinus*) and echidna (*Tachyglossus aculeatus*). RTOM genes encode reverse transcriptases and are expressed specifically in the testes of both the platypus and echidna. Such testis-specific reverse transcriptase genes have not been found in therian mammals, but their involvement in reproductive functions unique to monotremes has been previously discussed [24]. He also analyzed the genomes of birds and reptiles and found conservation of several protein-coding genes derived from LTR retrotransposons. These results indicate the diverse functions of LTR retrotransposon-derived proteins in vertebrates. Kuniaki Saito (National Institute of Genetics, Japan) discussed the subcellular localization of gypsy Gag and Env proteins in *Drosophila* ovarian somatic cell line OSC, in which Piwi-piRNA-dependent TE silencing occurs. Although the expression of ERV-derived proteins in the ovary has been reported previously [25], their functions and actions are not fully understood. To elucidate this in detail, Saito et al. generated monoclonal antibodies against gypsy Gag and Env proteins and found that Gag co-localized with Env in the cytoplasmic granules of Piwi-depleted OSCs. Env depletion dispersed Gag localization, whereas Gag depletion did not change Env localization. This implies the cooperative action of Gag and Env proteins in Piwi-depleted OSCs.

TEs also provide *cis*-regulatory elements that have altered gene expression during evolution. Many LTR retrotransposons in the human genome are primate-specific and introduce novel cis-regulatory elements into the lineage. While the transcriptional activity of LTRs is restricted by the host through various mechanisms, the nucleotide changes in LTRs associated with their activity and expansion during primate evolution remain largely unknown. Fumitaka Inoue (Kyoto University, Japan) et al. found that the MER11 family is one of the youngest simian-specific ERVs and is highly enriched with active epigenetic marks in human pluripotent stem cells. They analyzed the phylogenetic relationship between the MER11A/B/C subfamilies and classified them into multiple phyletic groups, which resolved the sequence and epigenetic heterogeneity of the MER11 subfamilies. To understand the functional divergence of MER11 sequences, massively parallel reporter assays (MPRAs) were used to systematically analyze MER11 sequence variants in human, chimpanzee, and macaque genomes to determine their regulatory activities [26, 27]. They found that transcription factors, such as ZIC and TEAD, are responsible for the regulatory activity of evolutionarily old MER11 phyletic groups. In contrast, evolutionarily young MER11 phyletic groups acquired POU- and SOX-binding motifs via nucleotide deletion(s) in the active core sequence. MPRA validated the correlation between POU and SOX motif acquisition and regulatory activity. Thus, this study delineates the functional divergence of MER11 along with nucleotide changes in primate genome evolution.

In Archaea, some tRNA genes have introns and some tRNA genes are split. Recently, an association between some intron-containing tRNA genes and TEs was reported. Akio Kanai (Keio University, Japan) et al. discovered disrupted tRNA genes in various species by computational analysis of their complete genomes. Notably, within the hyperthermoacidophilic archaeon *Caldivirga maquilingensis*, they found that tRNA^Gly^(TCC) and tRNA^Gly^(GCC) consist of three separate transcripts, a phenomenon termed “tri-split tRNA” [28]. Interestingly, the leader sequences, which serve as scaffolds for interlinking tri-split tRNAs within the cell, correspond to the tRNA intron regions found in closely related archaeal species, suggesting an evolutionary relationship between split tRNAs and intron-containing tRNAs. Moreover, certain tRNA introns transpose from one tRNA into multiple other tRNAs [29]. In this regard, such tRNA introns are considered as TEs. He also reported that the 16S and 23S rRNA genes of Candidate Phyla Radiation (CPR), a recently discovered bacterial phylum that is uncultured or challenging to cultivate, contain numerous introns. Interestingly, these introns are mobile group I or II introns, and some encode homing endonucleases [30], which shed light on the association between TEs and RNA genes in the prokaryotic world.

### TE expression in development and stress response

In preimplantation development in mice, the LTR retrotransposon MERVL is transiently expressed during zygotic gene activation (ZGA) at the 2-cell stage, which is essential for blastocyst formation [31]. In embryonic stem cells (ESCs) derived from the inner cell mass of blastocysts, MERVL expression is suppressed; however, MERVL-positive cells called 2-cell-like cells (2CLCs), spontaneously arise at a rate of approximately 0.1%. Cells expressing MERVL have been suggested to be totipotent. Hirotsugu Ishizu (Keio University) presented the mechanism underlying the emergence of 2CLCs through the reprogramming of mouse ESCs. By performing single-cell RNA-seq analysis of the cell population transitioning from ESCs to 2CLCs, they found that the retinoic acid signaling pathway plays an essential role in shaping the totipotent program. Indeed, treating ESCs with a retinoic acid analog activated the expression of MERVL and other 2-cell stage-specific genes, which promoted the conversion of the cells to 2CLCs. Elucidating the functions of MERVL regulated by the retinoic acid signaling pathway remains a task for future research.

In contrast to the expression analysis of long interspersed elements (LINEs) and LTR retrotransposons, the expression of short interspersed elements (SINEs), which are transcribed by RNA Pol III and are typically < 500-bp long, is difficult to analyze by deep sequencing because many copies reside within other transcripts such as mRNAs. Kenji Ichiyanagi (Nagoya University, Japan) recently established a deep sequencing method, melRNA-seq, to determine the RNA abundance, RNA length, and source loci of SINEs [32, 33]. They analyzed the expression of Alu SINE in human cells which revealed a 9-fold increase in Alu expression after heat shock treatment. The loci expressed under heat shock were predominantly located outside Pol II-transcribed genes and, interestingly, often contained binding motifs for transcription factors (TFs) involved in the immune response to viral infection. These results suggested that these TFs were involved in the upregulation of Alu during heat shock and viral infection. Because Alu RNA has been shown to regulate the activity of Pol II, this Alu-TF association may have biological significance and will be studied in the future.

## Data Availability

Not applicable.

## Abbreviations

2CLC: 2-cell-like cell
CPR: candidate phyla radiation
ESC: embryonic stem cell
EVE: endogenous viral-like element
ERV: endogenous retrovirus
LINE: long interspersed element
LTR: long terminal repeat
MAC: macronucleus
MIC: micronucleus
MPRA: massively parallel reporter assay
NIG: National Institute of Genetics
ORF: open reading frame
piRNA: PIWI-interacting RNA
RdDM: RNA-directed DNA methylation
RiCHE: RdDM-independent CH methylation establishment
RIL: recombinant inbred line
RNAi: RNA interference
SINE: short interspersed element
TE: transposable element
TF: transcription factor
TSD: target-site duplication
VLP: virus-like particles
WOX: WUSCHEL-related homeobox
ZGA: zygotic gene activation

## References

[1] Ichiyanagi K (2016). TE studies in Japan: the third Japanese meeting on host-transposon interactions. Mobile DNA-Uk..

[2] Ichiyanagi K, Saito K (2019). TE studies in Japan: the fourth Japanese meeting on host-transposon interactions. Mobile DNA-Uk..

[3] Ichiyanagi K, Saito K (2022). The fifth Japanese meeting on biological function and evolution through interactions between hosts and transposable elements. Mobile DNA-Uk.

[4] Kakutani T, To TK (2022). Crosstalk among pathways to generate DNA methylome. Curr Opin Plant Biol..

[5] Nishizawa Y, Inagaki S, Tarutani Y, Tominaga S, Toyoda A, To TK (2020). RNA interference-independent reprogramming of DNA methylation in *Arabidopsis*. Nat Plants..

[6] To TK, Yamasaki C, Oda S, Tominaga S, Kobayashi A, Tarutani Y (2022). Local and global crosstalk among heterochromatin marks drives DNA methylome patterning in *Arabidopsis*. Nat Commun..

[7] Ikeda Y, Nishihama R, Yamaoka S, Arteaga-Vazquez MA, Aguilar-Cruz A, Grimanelli D (2018). Loss of CG methylation in *Marchantia polymorpha* causes disorganization of cell division and reveals unique DNA methylation regulatory mechanisms of non-CG methylation. Plant Cell Physiol..

[8] Sasaki E, Gunis J, Reichardt-Gomez I, Nizhynska V, Nordborg M (2022). Conditional GWAS of non-CG transposon methylation in *Arabidopsis thaliana* reveals major polymorphisms in five genes. PLoS Genet..

[9] Hanasaki M, Masumoto H (2019). CRISPR/transposon gene integration (CRITGI) can manage gene expression in a retrotransposon-dependent manner. Sci Rep..

[10] Kawaoka S, Mitsutake H, Kiuchi T, Kobayashi M, Yoshikawa M, Suzuki Y (2012). A role for transcription from a piRNA cluster in de novo piRNA production. RNA..

[11] Shoji K, Umemura Y, Katsuma S, Tomari Y (2023). The piRNA cluster torimochi is an expanding transposon in cultured silkworm cells. PLoS Genet..

[12] Suzuki K, Morimoto M, Kondo C, Ohsumi Y (2011). Selective autophagy regulates insertional mutagenesis by the Ty1 retrotransposon in *Saccharomyces cerevisiae*. Dev Cell.

[13] Kataoka K, Mochizuki K (2015). Phosphorylation of an HP1-like protein regulates heterochromatin body assembly for DNA elimination. Dev Cell..

[14] Kataoka K, Mochizuki K (2017). Heterochromatin aggregation during DNA elimination in Tetrahymena is facilitated by a prion-like protein. J Cell Sci..

[15] Tomooka N, Naito K, Kaga A, Sakai H, Isemura T, Ogiso-Tanaka E (2014). Evolution, domestication and neo-domestication of the genus *Vigna*. Plant Genet Resour..

[16] Naito K, Wakatake T, Shibata TF, Iseki K, Shigenobu S, Takahashi Y, et al. Genome sequence of 12 *Vigna* species as a knowledge base of stress tolerance and resistance. bioRxiv. 2022. 10.1101/2022.03.28.486085.

[17] Fukai E, Yoshikawa M, Shah N, Sandal N, Miyao A, Ono S (2022). Widespread and transgenerational retrotransposon activation in inter- and intraspecies recombinant inbred populations of *Lotus japonicus*. Plant J..

[18] Kawasaki S, Nitasaka E (2004). Characterization of Tpn1 family in the Japanese morning glory: En/Spm-related transposable elements capturing host genes. Plant Cell Physiol..

[19] Hoshino A, Jayakumar V, Nitasaka E, Toyoda A, Noguchi H, Itoh T (2016). Genome sequence and analysis of the Japanese morning glory *Ipomoea nil*. Nat Commun..

[20] Ueda MT, Kryukov K, Mitsuhashi S, Mitsuhashi H, Imanishi T, Nakagawa S (2020). Comprehensive genomic analysis reveals dynamic evolution of endogenous retroviruses that code for retroviral-like protein domains. Mob DNA..

[21] Shoji H, Kitao K, Miyazawa T, Nakagawa S (2023). Potentially reduced fusogenicity of syncytin-2 in New World monkeys. FEBS Open Bio..

[22] Nakagawa S, Takahashi MU. gEVE: a genome-based endogenous viral element database provides comprehensive viral protein-coding sequences in mammalian genomes. Database (Oxford). 2016;2016:baw087. 10.1093/database/baw087 PMC4885607 27242033

[23] Kitao K, Shoji H, Miyazawa T, Nakagawa S (2023). Dynamic evolution of retroviral envelope genes in egg-laying mammalian genomes. Mol Biol Evol..

[24] Kitao K, Miyazawa T, Nakagawa S (2022). Monotreme-specific conserved putative proteins derived from retroviral reverse transcriptase. Virus Evol..

[25] Chalvet F, Teysset L, Terzian C, Prud'homme N, Santamaria P, Bucheton A (1999). Proviral amplification of the gypsy endogenous retrovirus of *Drosophila melanogaster* involves env-independent invasion of the female germline. EMBO J..

[26] Inoue F, Ahituv N (2015). Decoding enhancers using massively parallel reporter assays. Genomics..

[27] Gordon MG, Inoue F, Martin B, Schubach M, Agarwal V, Whalen S (2020). lentiMPRA and MPRAflow for high-throughput functional characterization of gene regulatory elements. Nat Protoc..

[28] Fujishima K, Sugahara J, Kikuta K, Hirano R, Sato A, Tomita M (2009). Tri-split tRNA is a transfer RNA made from 3 transcripts that provides insight into the evolution of fragmented tRNAs in archaea. Proc Natl Acad Sci U S A..

[29] Fujishima K, Sugahara J, Tomita M, Kanai A (2010). Large-scale tRNA intron transposition in the archaeal order *Thermoproteales* represents a novel mechanism of intron gain. Mol Biol Evol..

[30] Tsurumaki M, Saito M, Tomita M, Kanai A (2022). Features of smaller ribosomes in candidate phyla radiation (CPR) bacteria revealed with a molecular evolutionary analysis. RNA..

[31] Sakashita A, Kitano T, Ishizu H, Guo Y, Masuda H, Ariura M (2023). Transcription of MERVL retrotransposons is required for preimplantation embryo development. Nat Genet..

[32] Mori Y, Ichiyanagi K (2021). melRNA-seq for expression analysis of SINE RNAs and other medium-length non-coding RNAs. Mob DNA..

[33] Ichiyanagi T, Katoh H, Mori Y, Hirafuku K, Boyboy BA, Kawase M (2021). B2 SINE copies serve as a transposable boundary of DNA methylation and histone modifications in the mouse. Mol Biol Evol..

